# With or without the Mask: Age Differences in Perceived Trustworthiness and Health during the COVID-19 Pandemic

**DOI:** 10.3390/bs13030195

**Published:** 2023-02-22

**Authors:** Adolfo Di Crosta, Irene Ceccato, Emanuela Bartolini, Pasquale La Malva, Matteo Gatti, Eleonora D’Intino, Loreta Cannito, Anna Marin, Riccardo Palumbo, Nicola Mammarella, Alberto Di Domenico, Rocco Palumbo

**Affiliations:** 1 Department of Psychological, Health and Territorial Sciences, “G. d’Annunzio” University of Chieti-Pescara, 66100 Chieti, Italy; 2 Department of Neuroscience, Imaging and Clinical Sciences, “G. d’Annunzio” University of Chieti-Pescara, 66100 Chieti, Italy; 3 Center for Translational Cognitive Neuroscience, VA Boston Healthcare System, Boston, MA 02130, USA; 4 Center for Advanced Studies and Technology (CAST), “G. d’Annunzio” University of Chieti-Pescara, 66100 Chieti, Italy

**Keywords:** trust, health, face perception, coronavirus, ratings, trait impressions

## Abstract

The COVID-19 pandemic and the obligation to wear surgical face masks have affected social interactions. Wearing a mask can cause impairments in face identification, emotion recognition, and trait impressions. The present study investigated, during the COVID-19 period, age-related differences in perceived trustworthiness (Study 1) and health (Study 2) when viewing faces with or without masks. Younger (YAs, 18–35 years) and older (OAs, over 65 years) adults’ ratings were compared. Through a web-based platform, a series of neutral younger and older faces (YFs vs. OFs) were presented, on a computer screen, with or without a mask (Mask vs. No-Mask), and participants were asked to rate them on a 7-point scale. Furthermore, data collected during the pandemic (Mask and No-Mask conditions) were compared with ratings obtained before it (Pre-COVID condition). Perceived trustworthiness was lower in the No-Mask condition for both age groups compared to Mask and Pre-COVID conditions, which did not differ. For health ratings, no differences emerged for OAs between the conditions, whereas YAs’ ratings were lower in both the Mask and No-Mask conditions compared to the Pre-COVID condition. The fear of contracting COVID-19 affected both trustworthiness and health ratings. Wearing a surgical face mask affects trait impressions for YAs and OAs, partly due to the fear of COVID-19. Trait impressions are also influenced by the age of the face to be evaluated.

## General Overview

In March 2020, the World Health Organization (WHO) defined the COVID-19 outbreak as a global pandemic, providing guidelines to prevent the spreading of the virus [1,2,3]. However, disease containment measures, such as social distancing and wearing face masks, dramatically impacted human health and social interactions [4,5,6,7,8], in some cases leading to the exacerbation of clinical psychological symptoms [9]. Specifically, traits humans can infer from faces (i.e., trustworthiness, attractiveness, health) have a functional role in many aspects of everyday life [10,11]. Research conducted before the COVID-19 pandemic indicated that observing a partial face negatively impacted accurate face recognition and trait impressions [12]. The characteristics of the evaluator, as well as the face’s characteristics, also influence these processes [13,14].

In the COVID-19 pandemic context, early studies highlighted that, since the surgical face mask covers an important part of the face (about 60–70%), it could cause impairments in face identification, emotion recognition, and trait impressions [15]. Evidence showed that people wearing a face mask could be judged as more responsible and trustworthy because they give impressions of being more respectful of the new social norms [16]. Furthermore, Kamatani and colleagues [15] found that faces without a face mask were rated less healthy than those with a mask. The growing literature is progressively highlighting the role of individual differences in the psychological and behavioral responses to the COVID-19 emergency [17,18,19,20,21,22]. A study showed that people’s judgments of faces vary based on the utility associated with the face mask and their psychological distress levels [23].

To the best of our knowledge, no study has yet considered the impact of aging on this topic. However, the research highlighted the importance of considering age-related differences in the psychological reactions to the COVID-19 pandemic. For instance, older adults (OAs) exhibited a more positive attitude toward the emergency [24] and reported lower anxiety and depression symptoms, perceiving a lower risk of being infected compared to younger adults (YAs) [25].

The present work extends previous research on trait impressions from faces wearing a face mask by examining age-related differences. It investigates how ratings on perceived trustworthiness (Study 1) and health (Study 2) can vary based on a face mask’s presence or absence. We focused on these two traits because of their importance for social interactions in the COVID-19 emergency [26].

Studies conducted before the pandemic revealed age-related differences in trait impressions from faces. Palumbo and colleagues [10] examined YAs and OAs’ ratings of neutral faces, comparing younger (YFs) and older (OFs) faces. The results indicated that YAs, but not OAs, attributed to OFs higher warmth and lower competence. Previous studies indicated that OAs report more positive impressions of faces on health, hostility, and trustworthiness [27]. Furthermore, OAs showed greater positivity in evaluating the hostility of OFs compared to YFs, showing an “own-age bias” in trait impressions; that is, the tendency to prefer own-age over other-age faces [28]. These results have been explained within the theoretical frame of the Socioemotional Selectivity Theory [29], which states that OAs are more oriented toward pleasant emotions, tending to avoid negative stimuli (known as the “positivity effect”) due to their constrained temporal horizon [30,31,32].

A second contribution of the current research is the investigation of changes in trait impressions, comparing the ratings before and during the COVID-19 pandemic. Data from the study by Palumbo and colleagues [10] were used as a baseline to replicate the experimental design during the period of the COVID-19 pandemic, presenting the same faces with or without a face mask. This procedure allowed us to examine age differences in possible changes in trait impressions during the COVID-19 pandemic.

Finally, as previous studies indicated that the fear of the pandemic influences psychological responses and behaviors [33,34], it was tested whether individual differences in emotional states due to the emergency can modulate trait impressions.

## 1. Study 1—Trustworthiness

### 1.1. Introduction

Trustworthiness is related to those characteristics and conditions that facilitate the process by which a person is trusted [35]. Research showed that different factors can influence judgments of trustworthiness. For example, people judge a face as more trustworthy if it reflects the canons of the typical face: the more a face embodies typical features, the more reliable it is [36]. Other studies reported higher levels of trustworthiness when older faces (OFs) were evaluated compared to younger faces (YFs) [37]. This effect may be due to the stereotypical notion that OAs are more friendly and trustworthy than YAs [38].

A bulk of evidence underlined that older adults (OAs) evaluate faces as more trustworthy than younger adults (YAs) [27,39]. Finally, people vary in their interpersonal trust levels. This construct reflects the subjective belief that the sincerity or the goodwill of others can be relied upon [40].

Recent studies have examined the relationship between decreased trust and increased fear within the COVID-19 context [41,42]. Other studies have suggested increased perceived trustworthiness when people wear a face mask [43]. For instance, Olivera-La Rosa and colleagues [16] found that masked faces were judged more trustworthy than the same faces presented without a face mask. A study on interpersonal distance found that participants accepted greater physical closeness from virtual characters wearing a face mask than from characters without a mask, suggesting higher perceived trust when a mask is present [26].

Based on these premises, it was hypothesized that, during the COVID-19 pandemic, faces presented with a face mask (Mask condition) would be perceived as more trustworthy compared to the same stimuli presented without a mask (No-Mask condition). Considering the pandemic context, a reduction in trustworthiness ratings, in both Mask and No-Mask conditions, compared to those obtained before the COVID-19 outbreak (Pre-COVID condition), was hypothesized.

For age-related differences, it was hypothesized that OAs would rate faces as more trustworthy than YAs, irrespective of a face mask’s presence. Moreover, the authors expected that OFs would be evaluated as more trustworthy compared to YFs. If lower trustworthiness emerged during the COVID-19 pandemic, the authors expected OAs to show a smaller decrease in trustworthiness ratings compared to YAs due to the positivity effect.

### 1.2. Materials and Methods

#### 1.2.1. Participants

A power analysis, using G*Power 3.1.9.7, indicated that N = 24 (large effect size; f = 0.40) to N = 84 (small effect size; f = 0.10) participants in total are required to achieve 95% power when employing the traditional 0.05 criterion of statistical significance.

In total, 60 YAs (40 female; 18–35 years old) and 60 OAs (39 female; over 65 years old) were recruited (Table 1). Participants were recruited through word-of-mouth and social media, providing written informed consent. The Ethical Committee of the Department of Psychological, Health and Territorial Sciences, University “G. d’Annunzio”, approved the study. Participants received no monetary or other forms of compensation. Participants with a clinically significant problem of any of the following conditions have been excluded from the study: cerebrovascular disease, traumatic brain damage, a degenerative disease (e.g., frontotemporal dementia, Parkinson’s disease, Alzheimer’s disease), or any medical condition whose severity could significantly impair cognition (e.g., organ failure). For older adults, only participants who obtained a score indicative of healthy aging (cutoff score: 18) at the Montreal Cognitive Assessment (MoCA)-BLIND version were included [44]. The whole study was carried out in Italy during the phase of restrictive measures imposed to contain the COVID-19 pandemic between December 2020 and January 2021. Due to the impossibility of inviting participants to the laboratory, the procedure was conducted remotely on a web-based platform (Qualtrics). As in the study by Marini and colleagues [43], the platform was set to provide a single un-reusable link for each participant with an anti-ballot box stuffing to avoid multiple participations. Therefore, each participant could run the experiment directly from home on their laptop. Participants were instructed to perform the task and answer questionnaires while alone in a quiet room. Participants were asked to sit in front of a computer screen while maintaining a distance from the screen of about 60 cm for the entire duration of the task.

#### 1.2.2. Study Design

The present study replicated the procedure adopted by Palumbo and colleagues [10] by implementing a between-subjects design, which allowed us to maintain independent judgments on trustworthiness between the two experimental conditions. Participants were randomly divided into two groups, balancing age and gender. The first group, composed of 30 YAs and 30 OAs, was asked to rate faces presented with a face mask (Mask condition). A second group, composed of 30 YAs and 30 OAs, was asked to rate the same stimuli presented without a face mask (No-Mask condition). The original study by Palumbo and colleagues [10] involved 20 YAs and 20 OAs who judged the same faces without a face mask (Pre-COVID condition).

#### 1.2.3. Facial Stimuli

**Figure 1:**
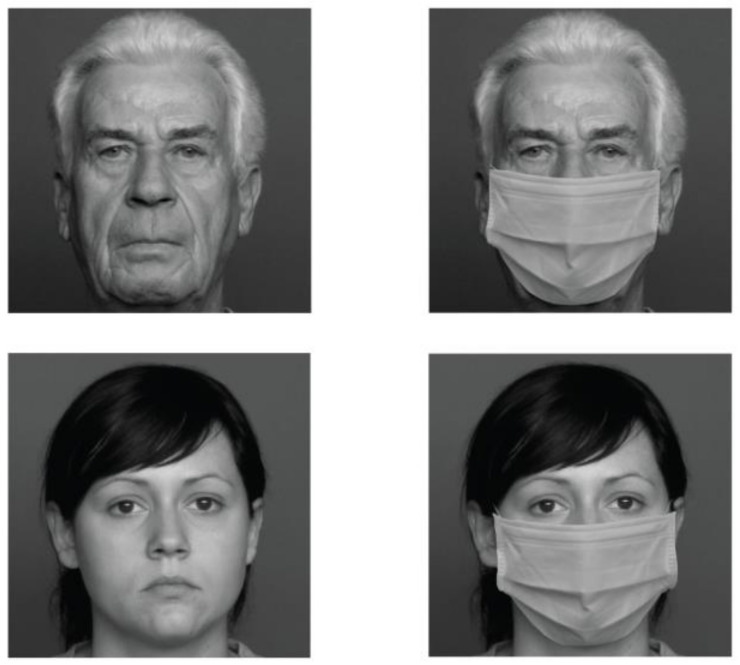
Examples of facial stimuli used in the studies. On the left are examples of original faces (OF and YF). On the right, the same stimuli wearing the face mask.

The facial stimuli used by Palumbo and colleagues [10] comprised 120 older and 120 younger neutral-expression faces, with men and women equally represented within each age group. These stimuli were extrapolated from the FACES [45] and they were presented in grayscale. The stimuli for the Mask condition were created by graphically applying a face mask to all the faces. Adobe Photoshop was used to cut out the picture of the mask and individually apply it to each stimulus (Figure 1).

#### 1.2.4. Fear of COVID-19 Questionnaire

The Fear of COVID-19 questionnaire was administered to measure fear and concerning beliefs related to the COVID-19 pandemic [9]. This questionnaire deals with the perceived probability of being infected (belief in contagion) and the severity of the contagion’s consequences (consequences of contagion). Responses were given on a Likert scale ranging from 0 (not at all) to 100 (extremely). The items and reliability values in the current study are reported in the Supplementary Material (Table S1).

#### 1.2.5. Lack of Trust Questionnaire

Participants’ levels of interpersonal trust were measured by combining items adapted from questionnaires commonly used in the literature [40,46,47]. Participants were asked to attribute a score to each of the eight items on a scale ranging from 1 (“I do not agree at all”) to 5 (“I agree absolutely). Higher scores reflected lower levels of trust. The Supplementary Material report the items and the reliability value (Table S2).

#### 1.2.6. Procedure

Socio-demographic information such as age, gender, and education level was collected. Education was measured on a scale ranging from 1 (i.e., elementary school) to 5 (i.e., postgraduate degree). For older adults, the MoCA-Blind was administered to control their cognitive status. Afterward, participants were randomly assigned to either Mask or No-Mask conditions, and the trustworthiness rating began. Participants had to rate each of the 240 faces for trustworthiness on a 7-point scale ranging from 1 (not at all trustworthy) to 7 (very trustworthy). Faces were presented randomly, one at a time for 2s each, after which the rating scale appeared. Once participants made their rating, a new face was shown. Finally, participants completed the Fear of COVID-19 and the lack of trust questionnaires.

#### 1.2.7. Statistical Analyses

**Figure 2:**
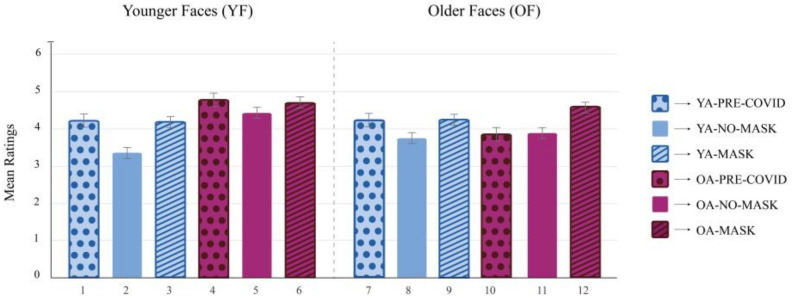
Repeated measures three-way ANOVA on trustworthiness ratings.

First, independent *t*-tests and one-way ANOVAs were conducted to compare the different samples of the present study for socio-demographic characteristics, results of questionnaires, and trustworthiness ratings (Table 1). Then, a 3 × 2 × 2 repeated-measures ANOVA was performed, with condition (Mask vs. No-Mask vs. Pre-COVID) and age group (YA vs. OA) as the between-subject factors and face age (YF vs. OF) as the within-subject factor. The trustworthiness ratings were entered as the dependent variable (Figure 2 and Table 2). Based on the ANOVA results, two hierarchical multiple regression analyses were conducted on the trustworthiness ratings obtained for the No-Mask condition in YAs and in OAs (Table 3). All analyses were performed in SPSS [48].

### 1.3. Results

A repeated-measures three-way ANOVA was conducted to examine the effect of condition (Mask vs. No-Mask vs. Pre-COVID), age group (YA vs. OA), and face age (YF vs. OF) on trustworthiness ratings. Post hoc analyses were performed using Tukey HSD tests (Figure 2). The main effect of the age group was statistically significant, *F*(1, 154) = 11.11, *p* < 0.01, η_p_^2^ = 0.07, indicating that OAs rated all faces as more trustworthy than YAs. The main effect of the condition was also significant, *F*(2, 154) = 10.63, *p* < 0.001, η_p_^2^ = 0.12. Post hoc analyses revealed that trustworthiness ratings in the No-Mask condition were lower than in the Mask condition, *p* < 0.001, and in the Pre-COVID condition, *p* = 0.011. No differences between Mask and Pre-COVID conditions emerged, *p* = 0.475. The interaction effect between age group and condition was not significant, *F*(2, 154) = 1.61, *p* = 0.202, η_p_^2^ = 0.02. The main effect of face age, included as a within-subjects factor, was statistically significant, *F*(1. 154) = 8.19, *p* < 0.01, η_p_^2^ = 0.05. Specifically, YFs were evaluated as more trustworthy than OFs, *p* = 0.005. The two-way interaction effect between face age and age group was significant, *F*(1, 154) = 27.57, *p* < 0.001, η_p_^2^ = 0.15, indicating that only OAs rated YFs as more trustworthy than OFs, *p* < 0.001, whereas no significant differences were detected for YAs between YF and OF ratings, *p* = 0.211. The two-way interaction effect between face age and condition was significant, *F*(2, 154) = 4.26, *p <* 0.05, η_p_^2^ = 0.05. Post hoc analyses revealed that YFs were evaluated as more trustworthy both in the Mask condition, *p* = 0.002, and in the Pre-COVID condition, *p* = 0.003, compared to the No-Mask condition. Furthermore, OFs were evaluated as more trustworthy in the Mask condition than in the No-Mask condition, *p* < 0.001, whereas no significant differences were detected compared to the Pre-COVID condition, *p* = 0.181. Finally, the three-way interaction effect between age group, condition, and face age was also significant, *F*(2, 154) = 4.35, *p* < 0.05, η_p_^2^ = 0.05, indicating a combined effect for these three factors on trustworthiness ratings. All the post hoc analyses are shown in Figure 2. Interestingly, YAs showed no significant differences in trustworthiness ratings in the Mask condition between YFs and OFs. Likewise, for OAs, there were no significant differences in trustworthiness ratings in the Mask condition between YFs and OFs. However, in the No-Mask condition, where trustworthiness ratings for all samples were lower compared to the other two conditions, YAs rated YFs as less trustworthy than OFs, whereas OAs showed the inverse pattern since they rated OFs as less trustworthy than YFs.

Two hierarchical multiple regression analyses were conducted to explore the potential factors contributing to the unexpected patterns obtained in the No-Mask condition. The first regression model (Model 1) investigated the predictors of trustworthiness ratings obtained in the No-Mask condition for YAs when YFs were evaluated. The second regression model (Model 2) investigated trustworthiness ratings obtained in the No-Mask condition for OAs when OFs were evaluated. For both regression models, control variables (gender and education) were entered as predictors in Step 1. In Step 2, the lack of trust questionnaire was entered. The two subscales of the Fear of COVID-19 questionnaire were entered in Step 3.

Concerning Model 1, the results showed that only the third step explained a significant amount of variance (see Table 3 for details). Specifically, the belief in contagion negatively predicted trustworthiness ratings, *p* = 0.006. Overall, the final model explained 37.9% of the variance in trustworthiness ratings of YAs in the No-Mask condition evaluating YFs.

Concerning Model 2, similar findings emerged: only the third step explained a significant amount of variance (see Table 3 for details), and only the belief in contagion subscale negatively predicted trustworthiness ratings, *p* = 0.012. Overall, the final model explained 30.0% of the variance in trustworthiness ratings of OAs in the No-Mask condition evaluating OFs. In both models, gender, education, and interpersonal trust did not significantly explain perceived trustworthiness.

### 1.4. Discussion

The results showed a significant difference in trustworthiness ratings during the COVID-19 pandemic between Mask and No-Mask conditions. Specifically, the results confirmed the hypothesis that the absence of a face mask during the COVID-19 pandemic lowered perceived trustworthiness. Comparing these data with those obtained before the COVID-19 pandemic, it is shown that faces were rated as less trustworthy in the No-Mask condition compared to the Pre-COVID condition. This result is in line with recent studies that documented the negative impact of the COVID-19 outbreak on trust levels [49]. There was no significant difference between Mask and Pre-COVID conditions. Therefore, the presence of the face mask produced perceived trustworthiness ratings comparable to those obtained before the COVID-19 outbreak, when people were not wearing face masks. Coherent with previous studies, mask wearers could be rated as more trustworthy because they gave impressions of taking the new social norms imposed by the COVID-19 context more seriously [16].

Our findings showed that perceived trustworthiness levels were influenced by age. Overall, OAs rated faces as more trustworthy compared to YAs. These data are in line with previous studies showing that OAs, expressing a positivity bias effect, generally give more positive ratings compared to YAs [50,51,52,53,54]. However, our hypothesis that, compared to YAs, OAs would show smaller differences in trustworthiness between ratings during and before the pandemic was not confirmed. Furthermore, some unexpected results were found considering the interaction between the face mask, participants’ age, and the age expressed by the facial stimuli. Indeed, YAs rated YFs not wearing a face mask as less trustworthy than OFs without a face mask. The opposite pattern emerged for OAs, who rated OFs without a face mask as less trustworthy than YFs not wearing a mask. However, previous studies conducted before COVID-19 showed the inverse pattern (i.e., an own-age bias preference) [28]. These results were explained based on the unprecedented health emergency context imposed by the COVID-19 pandemic. Indeed, it was found that trustworthiness ratings in both age groups were negatively influenced by individuals’ fear of contracting COVID-19. In our view, a possible explanation considers the social context characterizing the pandemic emergency. People were only allowed to meet other individuals by keeping interpersonal distance and wearing face masks. YAs may have judged their peers who do not wear face masks as more threatening to their health (and, therefore, less trustworthy) because it is them with whom they have most of their external contact. For the same reasons, OAs may have judged other older people who do not wear face masks as less trustworthy than differently aged (younger) people.

## 2. Study 2—Health

### 2.1. Introduction

Identifying unhealthy traits on a human face has a clear evolutionary benefit since it can allow one to maintain physical distance and avoid possible contagious diseases [55]. It has been demonstrated that OFs are perceived as less healthy than YFs, both by YAs and OAs, as the structural similarity of OFs to anomalous faces leads to general negative evaluations of their traits [56]. Research has also shown that, compared to YAs, OAs judge faces as healthier, likely reflecting the positivity effect [27].

Within the COVID-19 pandemic context, research has shown that the fear of contracting the virus has changed how people look at healthy and unhealthy faces [34]. Olivera-La Rosa and colleagues [16] found that mask wearers were perceived as less healthy than the same faces presented without a mask. Kamatani and colleagues [15] investigated the perceived health of faces with and without a surgical mask, comparing the results with those obtained before COVID-19. The findings indicated that mask-wearing faces were perceived as healthier during the COVID-19 pandemic compared to before the health emergency.

Based on the available findings, the authors expected a general reduction in health ratings during the COVID-19 emergency compared to before the pandemic outbreak. This reduction could be specifically associated with the individuals’ fear of contracting COVID-19. Based on the change in the purpose of face mask use during the COVID-19 pandemic, no significant differences between Mask and No-Mask conditions were expected.

It was hypothesized that OAs would judge faces as healthier than YAs based on the positivity effect. Moreover, it was expected that YFs would be evaluated as healthier than OFs, by both YAs and OAs. Furthermore, if a reduction in perceived health emerged during the COVID-19 pandemic compared to before the pandemic, OAs were expected to show a smaller decrease in health ratings compared to YAs.

### 2.2. Materials and Methods

A power analysis, using G*Power 3.1.9.7, indicated that N = 24 (large effect size; f = 0.40) to N = 84 (small effect size; f = 0.10) participants in total are required to achieve 95% power when employing the traditional 0.05 criterion of statistical significance

A total of 60 YAs (45 female; 18–35 years old) and 60 OAs (39 female; over 65 years old) were recruited (Table 4). The same criteria and procedure adopted for Study 1 were used. The study design, as well as the materials of this study, were identical to those adopted for Study 1.

#### 2.2.1. Procedure

The same between-subjects procedure in Study 1 was implemented. First, demographic information was collected. For the aims of Study 2, participants were asked to evaluate their own perceived health (i.e., health status) on a scale ranging from 1 (not at all healthy) to 5 (completely healthy). Afterward, they had to rate each of the 240 faces on a 7-point scale for health ranging from 1 (not at all healthy) to 7 (very healthy). Finally, participants completed the Fear of COVID-19 questionnaire.

#### 2.2.2. Statistical Analyses

**Figure 3:**
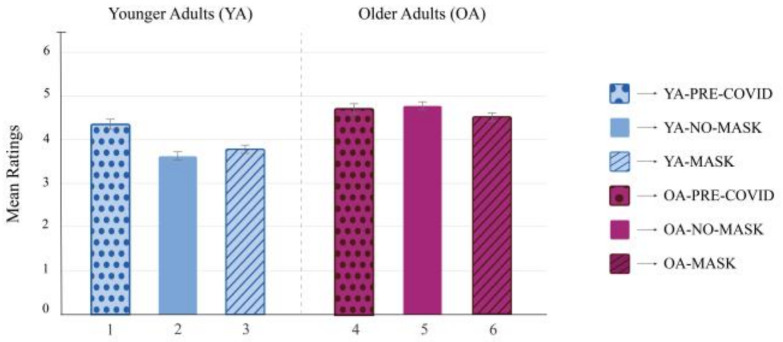
Repeated measures three-way ANOVA on health ratings.

First, independent *t*-tests and one-way ANOVAs were conducted to compare the different samples of the present study for socio-demographic characteristics, the results of questionnaires, and health ratings (Table 4). Then, a 3 × 2 × 2 repeated-measures ANOVA was performed, with condition (Mask vs. No-Mask vs. Pre-COVID) and age group (YA vs. OA) as the between-subject factors and face age (YFs vs. OFs) as the within-subject factor. The health ratings were entered as the dependent variable (Figure 3 and Table 5). Based on the ANOVA results, a multiple regression analysis was conducted on health (Table 6). All analyses were performed in SPSS [48].

### 2.3. Results

A repeated-measures three-way ANOVA was conducted to examine the effect of condition (Mask vs. No-Mask vs. Pre-COVID), age group (YA vs. OA), and face age (YF vs. OF) on health ratings. All post hoc analyses were performed using Tukey HSD tests. The results revealed a significant main effect of age group, F(1, 154) = 81.96, *p* < 0.001, ηp2 = 0.35, indicating that OAs rated all faces as healthier compared to YAs. The main effect of the condition was also significant, F(2, 154) = 7.80, *p* < 0.001, ηp2 = 0.09. Post hoc analyses revealed that health ratings in the Pre-COVID condition were higher than the Mask, *p* < 0.001, and No-Mask condition, *p* = 0.003. However, no differences in health ratings between the Mask and No-Mask conditions emerged, *p*= 0.822. The main effect of face age was significant, F(1, 154) = 552.61, *p* < 0.001, ηp2 = 0.78, indicating that YFs were evaluated as healthier than OFs, *p* < 0.001. The interaction between age group and condition was significant, F(2, 154) = 7.04, *p* < 0.01, ηp2 = 0.08. YAs evaluated faces as healthier in the Pre-COVID condition compared to both the Mask and No-Mask conditions (Figure 3). However, no statistical differences were found in OAs’ health ratings among the three experimental conditions. The two-way interaction effect between face age and age group was not significant, F(1, 154) = 2.89, *p* = 0.091, ηp2 = 0.02, nor was the two-way interaction effect between face age and condition, F(2, 154) = 0.23, *p* = 0.792, ηp2 = 0.01. Finally, the three-way interaction effect between age group, condition, and face age was also not significant, F(2, 154) = 2.16, *p* = 0.118, ηp2 = 0.03.

To explore the potential factors that contributed to the lower levels of health judgments during the COVID-19 pandemic (both in the Mask and No-Mask conditions) compared to before the pandemic, a hierarchical multiple regression analysis was performed on health ratings. Since no significant differences emerged between the Mask and No-Mask conditions in the ANOVA analysis, a single regression was performed by grouping together all the data collected during the COVID-19 pandemic. Control variables (including gender, age group, and education) were entered as predictors in Step 1. To further control for possible differences between the Mask and No-Mask conditions, the condition (Mask vs. No-Mask) was included as a factor in Step 2. In Step 3, health status was entered to examine the influence of participants’ subjective health on the health ratings of other people. Finally, in Step 4, the two subscales of the Fear of COVID-19 questionnaire were entered. The results showed that only the first and last steps explained significant amounts of variance (Table 6). Specifically, in Step 1, the effect of the age group was found to be significant, indicating that OAs provided higher health ratings compared to YAs. Step 4, in which the two subscales of the Fear of COVID-19 questionnaire were entered, explained 8% of the additional variance. Specifically, the belief in contagion scale, but not the consequences of contagion scale, significantly predicted health ratings. Gender, education, condition, and health status did not significantly predict perceived health from faces during the pandemic. Overall, the final model explained 58.0% of the variance in health ratings.

### 2.4. Discussion

The present study focused on age-related differences in people’s health judgments on faces wearing or not wearing a surgical mask, comparing ratings before and during the COVID-19 pandemic. The results revealed that health ratings were lower during the pandemic, regardless of the presence of a mask, compared to before the pandemic outbreak. However, no significant differences were found in health ratings between wearing or not wearing a mask during the COVID-19 pandemic. These data agree with our hypotheses and with recent studies focused on the differences in health impressions during the COVID-19 pandemic [15]. A plausible explanation is that since everyone was forced by law to wear a surgical face mask, this object ceased to symbolize illness, as it did before the pandemic [15]. This explanation is supported by our regression analysis, showing that the fear of contracting the disease, controlling for the presence/absence of the face mask, had an impact on health ratings.

For age-related differences, results showed that OAs rated faces as healthier compared to YAs, as hypothesized. Additionally, YFs were evaluated as healthier compared to OFs. These data are congruent with studies on face processing and trait formation conducted before the COVID-19 pandemic [56]. Finally, a substantial difference between YAs and OAs was found in health ratings in response to the COVID-19 pandemic. The results highlighted that only YAs had lower health judgments during the COVID-19 pandemic than before the virus outbreak. OAs’ health ratings did not differ between the COVID-19 and Pre-COVID conditions. According to our hypothesis, a possible explanation for why OAs did not show a decrease in health judgments due to the pandemic context can be related to the positivity effect.

## 3. Conclusions

The two studies investigated the impact of wearing a surgical face mask on people’s trait impressions from human faces in the COVID-19 context. For the first time, age-related differences in facial judgments were examined and the experimental stimuli comprised younger and older faces. Trustworthiness (Study 1) and health (Study 2) dimensions were analyzed for their importance in social interactions in the context of the COVID-19 pandemic. A further strength of the study is the possibility of comparing ratings obtained during the pandemic with those obtained before the COVID-19 outbreak by Palumbo and colleagues [10]. To summarize, compared to before the pandemic, the COVID-19 emergency caused lower levels of both trustworthiness and health judgments. In the pandemic period, not wearing a mask reduced perceived trustworthiness but not perceived health. Trustworthiness judgments were further influenced by age in terms of the observer (participant) and the observed (stimulus). Finally, individual differences in the fear of contracting COVID-19 partly account for the lower trustworthiness and health judgments during the pandemic.

In considering current results, some caveats need to be underlined. First, the study conducted before the COVID-19 pandemic lacks the condition in which the faces were wearing a mask. No doubt, the presence of this condition would have provided a better comparison with the data obtained during the pandemic. However, based on other studies [15], it seems reasonable to hypothesize that the presence of the mask on faces before the pandemic would lead to lower judgments of both trustworthiness and health dimensions because of its relationship with personal illness. Another limitation is the impossibility of inviting participants to the laboratory due to the COVID-19 restrictions measures. As in previous studies [43], a web-based platform has been set to avoid most of the associated technical problems and appositely instruct participants. However, since each participant completed the task on his/her personal computer, technical features (e.g., monitor refresh rate) were not controlled. Furthermore, as the survey was conducted online, we recruited a convenience sample that does not fully represent the population in Italy. Our findings did not extend to people who have no possibility or willingness to access the internet and carry out a web-based survey. This point may be particularly relevant for older adults. Finally, the present study considered the role of socio-demographic factors, of the individuals’ fear of contagion and the consequences of COVID-19 as well as the role of a general lack of trust in influencing face trait impressions. However, it was not possible to consider all the other factors that certainly influenced judgments on trustworthiness and health judgments (e.g., political position, media exposure, personality traits, etc.).

Notwithstanding these limitations, the present work increases our knowledge of how older and younger people experienced the COVID-19 emergency and how the presence of face masks differently affects their judgments of trustworthiness and health dimensions. Trait impression formation from faces is a crucial skill to navigate the social environment and relate to others and may impact psychological well-being. However, this study showed that wearing or not wearing a face mask in the context of the COVID-19 pandemic can hugely affect trait impressions. For instance, the emergency context changed the meaning of wearing a face mask. From a psychosocial point of view, this object ceased to symbolize a personal illness, as it did before the pandemic. Conversely, with the introduction of new social norms, not wearing a face mask has become a symbol of new, different negative social judgments (e.g., being less trustworthy, irresponsible, or disrespectful of the law). This unprecedented context may have contributed to generating a climate permeated by feelings of distrust, hatred, and fear toward each other. The present study seems to indicate that this social climate could affect more the relationships with peers, since they appear as more threatening and less responsible. The findings obtained from the present study can help to understand how social interactions changed during the COVID-19 pandemic due to different reactions to people’s faces and can help to highlight the climate that has arisen during the pandemic emergency. At a practical level, it is important to consider the psychological effects caused by the disruption of human social interaction during the COVID-19 pandemic. In this view, government institutions and policymakers should consider these aspects to design new, effective communication, even through the mass media, linked not only to the prevention of contagion and the respect of social norms but also aimed at restoring positive feelings of trust toward each other. At a theoretical level, the present results can provide a benchmark for future studies on changes in perceptions and judgments of others within emergency contexts. In conclusion, the present study, by shedding new light on changes in people’s trait impression due to the pandemic context, fits into the growing body of research that helps increase psychological and social preparedness in the face of future health emergencies.

## Figures and Tables

**Table 1:** Sample characteristics of Study 1. *M*, *SD*, and statistics separated for younger and older adults are reported.

	Younger Adults (YAs)				Older Adults (OAs)			
	Mask	No-Mask	Pre-COVID	Statistics	Mask	No-Mask	Pre-COVID	Statistics
	*M* (*SD*)	*M* (*SD*)	*M* (*SD*)		*M* (*SD*)	*M* (*SD*)	*M* (*SD*)	
Age	22.40 (4.12)	21.00 (2.49)	21.5 (1.70)	*F*(2,77) = 1.59, *p* = 0.210	73.90 (7.66)	72.17 (7.41)	72.30 (6.05)	*F*(2.77) = 0.51, *p* = 0.601
Education	3.03 (0.56)	3.10 (0.31)		*t*(58) = −0.58, *p* = 0.426	2.00 (1.02)	2.37 (1.25)		*t*(58) = −1.25, *p* = 0.200
Lack of trust	3.50 (0.63)	3.40 (0.54)		*t*(58) = 0.61, *p* = 0.472	3.48 (0.57)	3.31 (0.83)		*t*(58) = 0.88, *p* = 0.077
Belief in contagion	53.33 (21.83)	59.33 (18.92)		*t*(58) = −1.14, *p* = 0.260	45.92 (22.90)	49.93 (21.61)		*t*(58) = −0.70, *p* = 0.880
Consequences of contagion	37.62 (17.47)	42.86 (21.72)		*t*(58) = −1.03, *p* = 0.307	59.36 (20.56)	54.44 (24.24)		*t*(58) = 0.85, *p* = 0.212
Trustworthiness ratings	4.33 (0.60)	3.55 (0.58)	4.22 (0.60)	*F*(2,77) = 11.91, *p* < 0.001	4.65 (0.83)	4.16 (0.84)	4.32 (0.66)	*F*(2.77) = 2.88, *p* = 0.063

One-way ANOVAs were conducted for age and trustworthiness ratings comparing the three experimental groups. For the other variables, independent *t*-tests were conducted comparing only the Mask and No-Mask conditions.

**Table 2:** Pairwise comparisons for the repeated measures three-way ANOVA on trustworthiness ratings.

	1	2	3	4	5	6	7	8	9	10	11	12
*M* *(SE)*	4.22 (0.18)	3.35 (0.15)	4.19 (0.15)	4.78 (0.18)	4.43 (0.15)	4.70 (0.15)	4.22 (0.18)	3.75 (0.15)	4.25 (0.15)	3.86 (0.18)	3.90 (0.15)	4.60 (0.15)
**1**		0.01	0.999	0.562	0.999	0.664	0.999	0.68	0.999	0.957	0.965	0.906
**2**			0.003	<0.001	<0.001	<0.001	0.011	0.197	0.001	0.576	0.267	<0.001
**3**				0.332	0.994	0.392	0.999	0.606	0.999	0.953	0.96	0.737
**4**					0.939	0.999	0.554	0.001	0.512	<0.001	0.009	0.999
**5**						0.98	0.999	0.053	0.999	0.367	0.013	0.999
**6**							0.655	<0.001	0.603	0.016	0.007	0.999
**7**								0.688	0.999	0.959	0.967	0.901
**8**									0.396	0.999	0.999	0.003
**9**										0.863	0.863	0.893
**10**											0.999	0.065
**11**												0.038

**Table 3:** Summary of the regression analyses for variables predicting trustworthiness ratings in the No-Mask condition (Model 1 and Model 2).

	Model 1 YAs in the No-Mask Condition Evaluating YFs												Model 2 OAs in the No-Mask Condition Evaluating OFs											
	Step 1				Step 2				Step 3				Step 1				Step 2				Step 3			
	*B*	*SE*	*t*	*p*	*B*	*SE*	*t*	*p*	*B*	*SE*	*t*	*p*	*B*	*SE*	*t*	*p*	*B*	*SE*	*t*	*p*	*B*	*SE*	*t*	*p*
Gender	0.08	0.27	−0.32	0.752	−0.06	0.28	−0.23	0.823	0.00	0.24	0.01	0.994	0.11	0.26	0.44	.965	0.15	0.27	0.58	0.954	0.73	0.23	0.32	0.755
Education	0.48	0.43	1.12	0.271	0.48	0.44	1.10	0.281	0.38	0.38	1.00	0.326	−0.17	0.40	−0.42	.677	−0.17	0.41	−0.41	0.682	−0.26	0.36	−0.72	0.480
Lack of trust					−0.11	0.24	−0.45	0.659	0.06	0.21	0.30	0.766					−0.02	0.23	−0.08	0.934	0.13	0.21	−0.65	0.524
Belief in contagion									−0.02	0.01	−3.00	0.006									−0.02	0.01	−2.71	0.012
Consequences of contagion									−0.00	0.01	−0.22	.827									−0.00	0.01	−0.23	0.824
*R^2^*	0.05				0.05				0.38				0.01				0.01				0.31			
Δ*R^2^*	0.05				0.01				0.37				0.01				0.00				0.30			
*F* for Δ*R^2^*	0.63				0.20				63.19				0.09				0.01				5.19			
*p* for Δ*R^2^*	0.539				0.659				0.006				0.914				0.934				0.013			

Gender was coded 1 = male; 2 = female.

**Table 4:** Sample characteristics of Study 2. *M*, *SD*, and statistics separated for younger and older adults are reported.

	Younger Adults (YAs)				Older Adults (OAs)			
	Mask	No-Mask	Pre-COVID	Statistics	Mask	No-Mask	Pre-COVID	Statistics
	*M* (*SD*)	*M* (*SD*)	*M* (*SD*)		*M* (*SD*)	*M* (*SD*)	*M* (*SD*)	
Age	21.83 (3.69)	22.33 (4.44)	21.75 (1.83)	*F*(2,77) = 0.203, *p* = 0.817	72.90 (6.21)	72.40 (6.53)	72.30 (6.05)	*F*(2.77) = 0.513, *p* = 0.601
Education	3.17 (0.53)	3.10 (0.48)		*t*(58) = −0.510, *p* = 0.156	2.17 (1.26)	2.13 (1.22)		*t*(58) = 0.104, *p* = 0.918
Health Status	4.33 (0.55)	4.00 (0.95)		*t*(58) = 1.670, *p* = 0.037	3.60 (0.81)	3.40 (0.86)		*t*(58) = 0.928, *p* = 0.357
Belief in contagion	49.53 (22.70)	56.17 (19.50)		*t*(58) = −1.217, *p* = 0.552	43.33 (26.71)	45.67 (21.92)		*t*(58) = −0.371, *p* = 0.712
Consequences of contagion	44.12 (17.73)	45.29 (16.15)		*t*(58) = −0.268, *p* = 0.566	59.79 (25.51)	54.44 (24.24)		*t*(58) = 0.845, *p* = 0.212
Health ratings	3.79 (0.43)	3.63 (0.31)	4.36 (0.45)	*F*(2,77) = 21.413, *p* < 0.001	4.51(0.54)	4.78 (0.58)	4.73 (0.75)	*F*(2,77) = 1.564, *p* < 0.216

One-way ANOVAs were conducted for age and health ratings, comparing the three experimental groups. For the other variables, independent *t*-tests were conducted comparing only Mask and No-Mask conditions.

**Table 5:** Pairwise comparisons for the repeated measures three-way ANOVA on health ratings.

	1	2	3	4	5	6
*M* *(SE)*	4.36 (0.11)	3.63 (0.09)	3,79 (0.09)	4.73 (0.11)	4.78 (0.09)	4.51 (0.09)
1		<0.001	0.002	0.198	0.051	0.901
2			0.855	<0.001	<0.001	<0.001
3				<0.001	<0.001	<0.001
4					0.999	0.69
5						0.34

**Table 6:** Summary of the regression analysis predicting health ratings during the COVID-19 pandemic.

	Step 1				Step 2				Step 3				Step 4			
	*B*	*SE*	*t*	*p*	*B*	*SE*	*t*	*p*	*B*	*SE*	*t*	*p*	*B*	*SE*	*t*	*p*
Gender	−0.14	0.1	−1.397	0.165	−0.137	0.101	−1.365	0.175	−0.136	0.101	−1.353	0.179	−0.087	0.094	−0.93	0.354
Age group	0.019	0.002	9.324	<0.001	0.019	0.002	9.311	<0.001	0.018	0.002	8.698	<0.001	0.017	0.002	8.612	<0.001
Education	0.069	0.05	1.397	0.165	0.071	0.05	1.415	0.162	0.081	0.051	1.59	0.115	0.065	0.048	1.358	0.177
Condition					0.055	0.089	0.616	0.539	0.041	0.09	0.449	0.654	0.054	0.086	0.625	0.534
Health status									−0.056	0.058	−0.959	0.343	−0.08	0.054	−1.463	0.146
Belief in contagion													−0.008	0.002	−4.002	<0.001
Consequences of contagion													−0.003	0.002	−1.135	0.259
Δ*R^2^*	0.493				0.002				0.004				0.081			
*F* for Δ*R^2^*	37.61				0.38				0.92				10.78			
*p* for Δ*R^2^*	<0.001				0.539				0.340				<0.001			

Gender was coded 1 = male; 2 = female. Age group was coded 1 = YA; 2 = OA. Condition was coded 1 = Mask; 2 = No-Mask.

## Data Availability

The raw data supporting the conclusions of this article will be made available by the authors upon request, without undue reservation.

## Supplementary Materials

The following supporting information can be downloaded at: https://www.mdpi.com/article/10.3390/bs13030195/s1. Table S1. Items and reliability for the Fear for COVID-19 questionnaire; Table S2. Items and reliability of the Lack of trust questionnaire.
