# Supplementary material for: With or without the Mask: Age Differences in Perceived Trustworthiness and Health during the COVID-19 Pandemic
Source: Behav Sci (Basel). 2023 Feb 22;13(3):195. doi: 10.3390/bs13030195 (PMC10045182; doi:10.3390/bs13030195)
Supplement: Supplementary file 1 [file behavsci-13-00195-s001.zip › behavsci-2234909-supplementary.pdf]

Supplementary Materials

With or without the Mask: Age Differences in Perceived Trustworthiness and Health during the COVID-19 Pandemic

Table S1. Items and reliability for the Fear for COVID-19 questionnaire.

		Cronbach's Alpha	
**Item**	**Subscales**	**Study 1**	**Study 2**
I often thought I was infected with the virus<br>I think I could be infected with the virus in the future<br>I think that a dear or close person to me could potentially be infected with the virus<br>I think that a dear or close person to me could potentially be infected with the virus in the future	**Belief of contagion**	.765	.799
I think that a person infected with the virus could recover<br>I think that a person infected with the virus could die<br>I think it is probable that I would recover after being infected with the virus<br>I think that being infected with the virus could be lethal for me	**Consequences of contagion**	.809	.778

Table S2. Items and reliability of the Lack of trust questionnaire.

	Cronbach Alpha
**Item**	**Study 1**
If given a chance, most people try to take advantage of you.<br>Most people are too busy looking out for themselves to be helpful.<br>You can't trust stranger anymore.<br>I never rely on other people.<br>Most people can be trusted.*<br>You can't be too careful in dealing with people.<br>People would try to be fair.*<br>Most of the time people try to be helpful.*	.747

Note. *reversed items.
